# Beneficial Effects of Resistance Exercise on Glycemic Control Are Not Further Improved by Protein Ingestion

**DOI:** 10.1371/journal.pone.0020613

**Published:** 2011-06-20

**Authors:** Leigh Breen, Andrew Philp, Christopher S. Shaw, Asker E. Jeukendrup, Keith Baar, Kevin D. Tipton

**Affiliations:** 1 School of Sport and Exercise Sciences, University of Birmingham, Birmingham, United Kingdom; 2 Department of Kinesiology, McMaster University, Hamilton, Ontario, Canada; 3 Functional Molecular Biology Lab, Neurobiology, Physiology and Behaviour, University of California Davis, Davis, California, United States of America; 4 Department of Sports Studies, University of Stirling, Stirling, United Kingdom; University of Las Palmas de Gran Canaria, Spain

## Abstract

**Purpose:**

To investigate the mechanisms underpinning modifications in glucose homeostasis and insulin sensitivity 24 h after a bout of resistance exercise (RE) with or without protein ingestion.

**Methods:**

Twenty-four healthy males were assigned to a control (CON; *n* = 8), exercise (EX; *n* = 8) or exercise plus protein condition (EX+PRO; *n* = 8). Muscle biopsy and blood samples were obtained at rest for all groups and immediately post-RE (75% 1RM, 8×10 repetitions of leg-press and extension exercise) for EX and EX+PRO only. At 24 h post-RE (or post-resting biopsy for CON), a further muscle biopsy was obtained. Participants then consumed an oral glucose load (OGTT) containing 2 g of [U-^13^C] glucose during an infusion of 6, 6-[^2^H_2_] glucose. Blood samples were obtained every 10 min for 2 h to determine glucose kinetics. EX+PRO ingested an additional 25 g of intact whey protein with the OGTT. A final biopsy sample was obtained at the end of the OGTT.

**Results:**

Fasted plasma glucose and insulin were similar for all groups and were not different immediately post- and 24 h post-RE. Following RE, muscle glycogen was 26±8 and 19±6% lower in EX and EX+PRO, respectively. During OGTT, plasma glucose AUC was lower for EX and EX+PRO (75.1±2.7 and 75.3±2.8 mmol·L^−1^∶120 min, respectively) compared with CON (90.6±4.1 mmol·L^−1^∶120 min). Plasma insulin response was 13±2 and 21±4% lower for EX and CON, respectively, compared with EX+PRO. Glucose disappearance from the circulation was ∼12% greater in EX and EX+PRO compared with CON. Basal 24 h post-RE and insulin-stimulated PAS-AS160/TBC1D4 phosphorylation was greater for EX and EX+PRO.

**Conclusions:**

Prior RE improves glycemic control and insulin sensitivity through an increase in the rate at which glucose is disposed from the circulation. However, co-ingesting protein during a high-glucose load does not augment this response at 24 h post-exercise in healthy, insulin-sensitive individuals.

## Introduction

Metabolic complications, such as insulin resistance and Type II diabetes, represent a major individual and public health burden [1] and are associated with the presence of obesity and physical inactivity [2], [3], [4]. Suppressed pancreatic β cell insulin production [5] and impaired glucose uptake in skeletal muscle [6], [7], are major contributors to hyperglycemia, which eventually leads to Type II diabetes. Insulin stimulates the uptake of glucose from the circulation into many body tissues, of which, skeletal muscle accounts for ∼75–80% [8], [9]. Insulin-stimulated glucose uptake in skeletal muscle occurs via insulin-dependent signalling that promotes glucose transporter (GLUT4) translocation to the cell membrane. Even though pharmacologic approaches are available to manage Type II diabetes, The American Diabetes Association states that “physical activity and dietary modifications are central to the management and prevention of Type II diabetes” and when medications are used to control Type II diabetes, “they should augment lifestyle improvements, not replace them” [10].

Skeletal muscle contraction can effectively enhance glucose uptake independent of insulin. Importantly, these properties are preserved in individuals with Type II diabetes [11], [12]. A number of studies have demonstrated that aerobic exercise improves glycemic control and insulin sensitivity [13], [14], [15], [16]. More recently, evidence has emerged to suggest that a single bout of resistance exercise (RE) improves whole-body insulin sensitivity [17], [18], [19] and glycemic control [20], with the beneficial effects of RE on glycemic control and insulin sensitivity noted primarily between 12–24 h post-RE. However, not all studies demonstrate a beneficial effect of RE on glucose metabolism [21], [22].

Insulin regulates GLUT4 translocation from an intracellular location to the cell surface via a well-described pathway involving protein kinase B (Akt) and the recently discovered GTPase, Akt substrate of 160 kDa (AS160/TBC1D4) [23]. Although enhanced glucose uptake with acute RE is thought to occur via mechanisms that bypass proximal insulin-signalling intermediates, such as Akt [24], [25], [26], AS160/TBC1D4 appears to act as a point of convergence for insulin- and contraction-stimulated glucose transport in skeletal muscle [23], [27], [28]. Recently, basal and insulin-stimulated AS160/TBC1D4 phosphorylation was shown to increase in the acute post-exercise phase in humans [23], [29] and remain elevated for up to 27 h post-exercise in rodents [30]. However, whether AS160/TBC1D4 remains elevated in the 24 h post exercise period has not been investigated in humans.

In addition to RE, dietary modifications that acutely raise endogenous insulin secretion, represent a clinically relevant strategy to improve blood glucose homeostasis in Type II diabetes. A number of recent studies suggest that co-ingesting protein and/or amino acids with carbohydrate induces a greater insulin release than the ingestion of either macronutrient alone [31], [32], [33], [34]. Indeed, the rise in plasma insulin with the ingestion of frequent, small boluses of carbohydrate plus protein, blunts the prevailing glucose response in Type II diabetics [35], [36], primarily due to an increase in the rate of glucose disposal from the circulation [35]. Thus, in persons with Type II diabetes, co-ingesting protein with each main meal may be an effective strategy to acutely lower postprandial glucose excursions [32], [37]. The combined effect of protein ingestion and RE has largely been considered in the context of muscle hypertrophy [38], [39]. However, the hypothesis that post-RE protein ingestion, provided in a physiologically dose, acutely enhances glucose uptake has not been investigated in humans.

Therefore, we hypothesized that the beneficial effects of RE on post-prandial blood glucose homeostasis at 24 h post-RE would be explained by contraction-dependant alteration in rates of glucose disposal. Furthermore, we expected that the increase in glucose disposal with prior exercise would occur in parallel to increased activation of the Akt/AS160/TBC1D4 signaling cascade. Finally, we postulated that co-ingestion of protein during an oral glucose load would elevate the insulin response and augment the glucose-lowering effect observed following RE.

## Methods

### Participants

Twenty-four untrained, recreationally active, healthy males were recruited through advertisements to participate in the study. Individuals who were engaged in regular structured resistance or endurance training, defined as ≥2 training sessions per week of 60 mins or more, were ineligible to participate. All testing visits were completed within a 3-week period. The purpose and methodology of the study were clearly explained to the participants. All participants gave their informed consent prior to taking part in the study and were deemed healthy based on their response to a general health questionnaire. The experimental protocol was approved by the NHS Birmingham East, North & Solihull Research Ethics Committee (Rec No: 09/H1206/102).

### Study design

In a randomized, parallel designed study, participants were assigned to either a non-exercise control (CON; *n* = 8), exercise only (EX; *n* = 8) or exercise plus protein (EX+PRO; *n* = 8) condition. A parallel study design, in contrast to a crossover design, was chosen as ethical requirements meant that limitations were imposed on the number of muscle biopsy samples we could obtain per participant. Participant characteristics are presented in Table 1. Following a preliminary assessment of maximal lower-limb strength, participants reported to the laboratory after an overnight fast, on two consecutive mornings. On the first morning, a resting muscle biopsy sample was obtained, thereafter EX and EX+PRO performed an intense lower-limb resistance workout. A second biopsy was obtained immediately post-exercise for EX and EX+PRO only. Twenty-four hours later another muscle biopsy was obtained, after which, participants completed an oral glucose tolerance test (OGTT). Participants assigned to EX+PRO co-ingested protein with the OGTT to determine whether the addition of protein augmented the impact of resistance exercise on glucose metabolism. During OGTT, dual isotopic glucose tracers were utilized and frequent blood samples obtained over 2 h to determine glucose kinetics and insulin sensitivity. A final muscle biopsy was obtained at the end of the 2 h OGTT to examine the phosphorylation of contraction- and insulin-mediated signalling intermediates.

**Table 1:** Characteristics of participants in each group.

Parameter	CON (*n* = 8)	EX (*n* = 8)	EX+PRO (*n* = 8)
**Age (y)**	22±3	20±3	22±6
**Weight (kg)**	77.0±6.3	79.7±14.5	75.6±13.1
**BMI (kg·m^−2^)**	24±2.3	25.1±3.9	23.5±4.8
**LP 1RM (kg)**	200±44	212±50	206±60
**LP 1RM (kg·BM^−1^)**	5.7±0.8	5.9±1.8	6.0±1.3
**LE 1RM (kg)**	111±22	121±21	115±29
**LE 1RM (kg·BM^−1^)**	3.5±1.2	3.7±0.7	3.4±0.6
**Exercise volume (kg)**	^	18,314±2154	18,277±2406

CON: resting control group, EX: exercise only group, EX+PRO: exercise plus protein group. BMI: body mass index, 1RM: one-repetition maximum, LP: leg press, LE: leg extension. Exercise volume is defined as number of repetitions×number of sets×weight lifted. Values are presented as means ± SD.

### Preliminary testing

#### Body Mass

A digital scale was used to determine body mass to the nearest 0.1 kg. Participant weight and height were recorded in exercise clothing without shoes on. This was repeated prior to each of the two testing visits to ensure body mass remained stable throughout testing.

#### Maximal Strength

Bilateral 1 Repetition Maximum (1RM) was determined for leg press and leg extension exercises (Cybex VR/3). After warming up at a self selected resistance, the load was set at a level designed to allow the subject to perform at least two, but less than ten repetitions before failure. This estimation protocol of 1RM was designed to minimise the number of attempts necessary to determine 1RM [40]. After each successful lift the load was increased by 2.5–5 kg until failure to complete two repetitions. Between each successive attempt a 2 min rest period was allowed. A repetition was considered valid if the participant used proper form and was able to complete the entire lift in a controlled manner without assistance.

#### Diet and Physical Activity Control

Participant diet was standardized for the entire 48 h testing period. During preliminary testing participants completed a 3-day food diary, representative of their average week (two week days and one weekend day). A questionnaire of food preferences was also completed by participants. Using an on-line diet planner (Weight Loss Resources) the total energy and macronutrient content of each of the 3-days was estimated. Food parcels were provided to each participant with a total energy and macronutrient intake equivalent to their average habitual intake. Thus, participant diet was not manipulated during the study. Participants were instructed to consume only the food provided for them over the two-day testing period (i.e. 24 h prior to Day 1 and during Day 1). There was no difference in total energy intake or macronutrient composition of the food parcels for CON, EX and EX+PRO (Table 2).

**Table 2:** Participant habitual dietary intake and macronutrient composition.

	CON (*n* = 8)	EX (n = 8)	EX+PRO (n = 8)
**Daily energy intake (kJ)**	9,353±469	9,487±691	10,345±452
**Carbohydrate (%)**	71.1±6.6	66.2±3.9	69±7.1
**Protein (%)**	15.8±3.0	18.2±3.3	17.9±6.5
**Fat (%)**	13.1±3.1	15.6±0.4	13.1±4.8

Groups as per Table 1. Values are presented as means ± SEM.

#### Physical activity control

Participants were instructed to maintain normal habitual activities of daily living but to refrain from any strenuous activity for 48 h prior to reporting to the laboratory on Day 1. After completion of testing on Day 1, participants were also instructed to refrain from strenuous activity prior to returning the following morning on Day 2.

### Treatment trials

#### Day 1 - Exercise/Control Trial

Participants reported to the Human Performance Laboratory between 0600 and 0700 h after an overnight fast, 7–14 days after preliminary strength tests. After resting in a supine position for 30 min a cannula was inserted into an antecubital forearm vein and a resting blood sample was obtained for analysis of background isotopic enrichment. Thereafter, the lateral portion of one thigh was prepared under local anaesthetic (1% Lidocaine) and a 5-mm Bergstrom biopsy needle was used to extract a muscle biopsy sample from the *vastus lateralis* muscle. The biopsied leg was bandaged and EX and EX+PRO were instructed to complete a bout of intense lower-limb resistance exercise, whereas CON were permitted to consume the standardized breakfast and leave the laboratory. For EX and EX+PRO a second biopsy sample was obtained post-exercise (6±1 min) 1 cm distal from the resting biopsy. For EX and EX+PRO both biopsy incisions were made at rest to allow the post-exercise biopsy sample to be obtained as quickly as possible. The order of biopsied leg was counterbalanced between groups. Biopsy samples were blotted and freed of any visible fat and connective tissue, frozen in liquid nitrogen (within ∼60 s of being taken from the muscle) and stored at −80°C until further analysis.

The resistance exercise bout consisted of a standardized warm-up on a leg-press machine (12×50% 1RM+10×60% 1RM+8×70% 1RM+2×75% 1RM) followed by 8 sets of 10 bilateral repetitions at 75% 1RM. Participants then completed 8 sets of 10 bilateral repetitions on a leg-extension machine at 75% 1RM. The exercise protocol was chosen to match that used by Koopman and colleagues [17], in order to achieve an exercise effect on glycemic control. In the event that a participant failed to complete all 10 repetitions in a set, the weight was decreased by 2.5–5 kg for the following set. Failure was defined as the point at which the exercise could not be completed or technique failed. Participants were instructed on proper lifting cadence using a metronome set to 50 beats min^−1^, which corresponded to 1 s concentric muscle action, 0 s pause and a 1 s eccentric muscle action. Strong verbal encouragement was given throughout exercise. Between-set rest intervals of 2 min were given and participants completed the exercise bout in 45±3 min. Participants were permitted to consume water *ad libitum* throughout Day 1.

#### Day 2 - Infusion Trial

**Figure 1:**
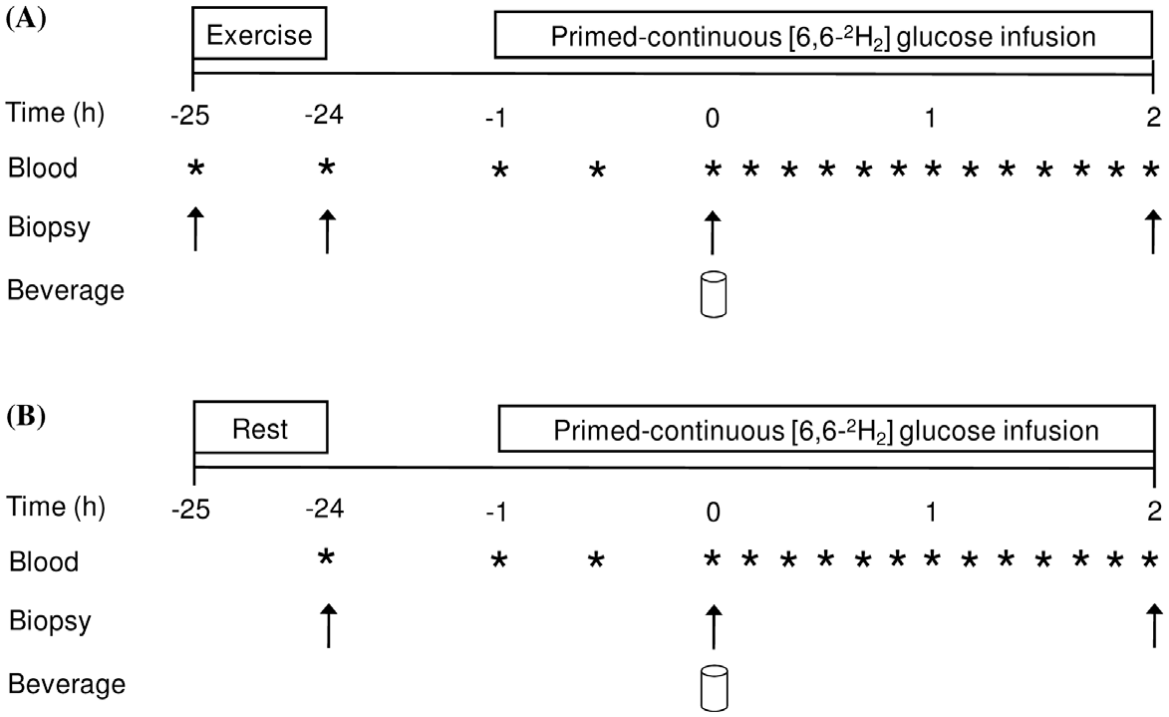
Schematic diagram of the study protocol. (A) Indicates protocol for exercise only and exercise plus protein participants. (B) Indicates protocol for non-exercised control participants.

Participants returned to the laboratory the following morning between 0600 and 0700 h after an overnight fast. A schematic diagram of the study protocol is presented in Figure 1. A cannula was inserted into the antecubital vein of one arm for the infusion of a stable isotopic tracer. A second cannula was inserted into a hand vein of the opposite arm and a resting venous blood sample obtained. At a time-point corresponding to ∼23 h post-exercise for EX and EX+PRO a primed infusion of 6, 6-[^2^H_2_] glucose (Cambridge Isotope Laboratories, MA, USA) was initiated (prime: 13.5 µmol.kg^−1^; infusion: 0.35 µmol.kg^−1^.min^−1^) and continued for ∼180 min. For CON, the infusion was initiated at a time-point corresponding to ∼23 h after the resting biopsy on Day 1. Approximately 60 min into the infusion (∼24 h post-exercise or resting biopsy) a muscle biopsy sample was obtained from the *vastus lateralis* of the opposite leg to that sampled the previous day. Immediately after the muscle biopsy was obtained CON and EX consumed an oral glucose load (oral glucose tolerance test; OGTT) described below. EX+PRO consumed the same glucose load plus additional protein. The time at which the beverage was completely consumed was considered *t = 0*, thereafter participants rested in the supine position and venous blood samples were obtained every 10 min until *t = 120*. A final muscle biopsy sample was obtained at *t = 120* from a separate incision (∼26 h post-exercise or resting biopsy). Water intake was restricted during Day 2 to ensure participants consumed only the treatment beverage.

### Treatment beverages

Sixty minutes into the infusion on Day 2, participants ingested 73 g of glucose (80.3 g dextrose monohydrate when corrected for water content) plus an additional 2 g of [U-^13^C] glucose (99%, Cambridge Isotope Laboratories, MA, USA) to determine the contribution of exogenous glucose production to the total rate of appearance of glucose. Thus, participants ingested a total of 75 g of glucose. Participants assigned to EX+PRO ingested an additional 25 g of whey protein with the 75 g glucose load. Glucose was provided in the form of dextrose monohydrate obtained from Roquette™ (Lestrem, France) and intact whey protein (Volactive ultrawhey 90) was a generous gift from Volac™ (Royston, UK). The amino acid content of the protein was (in percent content, wt∶wt): Ala, 5; Arg, 2.1; Asp, 11; Cys, 2.2; Gln, 18.1; Gly, 1.4; His, 1.7; Ile, 6.4; Leu, 10.6; Lys, 9.6; Met, 2.2; Phe, 3; Pro, 5.5; Ser, 4.6; Thr, 6.7; Trp, 1.4; Tyr, 2.6; and Val, 5.9. Both treatments were consumed in water in a total volume of 300 mL. Treatments were not matched for flavour or appearance due to the parallel study design. Participants were instructed to consume the treatment beverage within 2 min.

### Analyses

#### Blood analytes

Blood was collected in EDTA-containing tubes and spun at 3,500 rpm for 15 min at 4°C. Aliquots of plasma were then frozen and stored at −80°C until later analysis. Plasma glucose concentration was analyzed using an ILAB automated analyzer (Instrumentation Laboratory, Cheshire, UK). Plasma insulin concentration was analyzed using a commercially available ELISA kit (IBL International, Hamburg, Germany) following the manufacturer's instructions. The enrichment of [^13^C] and [^2^H_2_] glucose in plasma was determined by gas chromatography-mass spectrometry (model 5973; Hewlett Packard, Palo Alto, CA). Derivatization was carried out with butane boronic acid in pyridine and acetic anhydride. The glucose derivative was quantified by selected ion monitoring at mass-to-charge ratios (*m*/*z*) 297, 299 and 303 for [^12^C]-, [^2^H_2_]- and [U-^13^C] glucose, respectively. Two sets of enriched standards were measured containing known amounts of [^2^H_2_]- and [U-^13^C] glucose. By establishing the relationship between the enrichment of the glucose standards, the enrichment in plasma samples was determined.

#### Western blots

Muscle biopsy samples (∼40 mg) were powdered on dry ice under liquid nitrogen using a mortar and pestle. Approximately 25 mg of powdered muscle was homegenized in lysis buffer (50 mM Tris pH 7.5; 250 mM Sucrose; 1 mM EDTA; 1 mM EGTA; 1% Triton X-100; 1 mM NaVO4; 50 mM NaF; 0.50% PIC) using a hand-held homogenizer (PRO200, UK). Samples were shaken at 4°C for 30 min (12,000 rpm), centrifuged for 5 min at 6,000 g and the supernatant removed for protein determination. Protein concentration was determined using the DC protein assay (Bio Rad, Hertfordshire, UK). Equal aliquots of protein were boiled in Laemmli sample buffer (250 mM Tris-HCl, pH 6.8; 2% SDS; 10% glycerol; 0.01% bromophenol blue; 5% β-mercaptoethanol) and separated on SDS polyacrylamide gels (10–12.5%) for 1 h at 58 mA. Following electrophoresis; proteins were transferred to a Protran nitrocellulose membrane (Whatman, Dassel, Germany) at 100 V for 1 h. Samples from each of the three experimental conditions were loaded onto the same gel, such that each gel contained eleven samples (3 from CON, 4 from EX and 4 from EX+PRO). Total protein and phosphorylated protein were run concurrently on back-to-back gels using the same samples. The membranes were incubated overnight at 4°C with the appropriate primary antibody. The following morning, the membrane was rinsed in wash buffer (TBS with 0.1% Tween-20) three times for 5 min. The membrane was then incubated for 1 h at room temperature within wash buffer containing the appropriate secondary antibody, either horseradish (HRP)-linked anti-mouse IgG (New England Biolabs, 7072; 1∶1,000) or anti-rabbit IgG (New England Biolabs, 7074; 1∶1,000). The membrane was then cleared in wash buffer three times for 5 min. Antibody binding was detected using enhanced chemiluminescence (Millipore, Billerica, MA). Imaging and band quantification were carried out using a Chemi Genius Bioimaging Gel Doc System (Syngene, Cambridge, UK). Insufficient muscle tissue in 6 subjects (2 per group) meant that western blot analyses were determined for 18 participants (*n* = 6 per group). The primary antibodies used were total Akt (CAT), Akt^ser473^ (Cell signalling 3787), PAS-AS160/TBC1D4 (Cell Signalling) and total AS160/TBC1D4, a generous gift from Prof. Grahame Hardie, University of Dundee. Total p70S6K and phospho p70S6K^Thr389^ were from Santa Cruz (11759/7984R) and total PRAS40 and phospho PRAS40^Thr246^ were from Cell Signalling (2610).

#### Immunoprecipitations

Endogenous AS160/TBC1D4 and p70S6K proteins were immuno-precipitated (IP) overnight at 4°C with 0.8 µg of AS160/TBC1D4 or p70S6K antibodies in a mix of protein G-agarose beads (Millipore, Glostrup, DK) and lysate (600 µg). The following day immunocomplexes were washed three times in homogenization buffer and three times in TNE (10 mM Tris, pH 7.5, 150 mM NaCl, 10 mM EDTA and 0.1 mM Na_2_VO_4_). The immunocomplexes were re-suspended in 50 µL of 1×Laemmli sample buffer and boiled for 5 min (100°C) upon which they were subjected to western blotting as previously described.

#### Muscle glycogen measurement

Powdered muscle (∼20 mg) was hydrolyzed in 250 µl of 2 M HCl by heating at 95°C for 3 h. The solution was neutralized with 250 µl 2 M NaOH and the resulting free glycosyl units were assayed spectrophotometrically using a hexokinase-dependant assay kit (Glucose HK, ABX diagnostics, UK) against glucose standards of known concentrations [41].

### Calculations

#### Insulin sensitivity

Plasma glucose and insulin concentrations during the 120 min OGTT were used to determine the whole-body insulin sensitivity index (ISI) according to the following equation of Matsuda [42]:
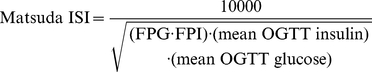
(1)Where FPG is the fasting plasma glucose concentration, FPI is the fasting insulin concentration and 1000 represents a constant that allows numbers between 1 and 10 to be obtained.

Post-absorptive insulin sensitivity was also estimated by the homeostasis model assessment (HOMA-IR) index which is calculated by dividing the product of FPG and FPI by 22.5 [43].

#### Glucose kinetics

From the [^2^H_2_] glucose tracer, the total R_a_ (*Eq. 2*) and R_d_ (*Eq. 3*) of glucose were calculated with the single-pool non-steady-state equations of Steele [44] as modified for use with stable isotopes [45]. Total R_a_ represents the splanchnic R_a_ of glucose from ingested glucose, the liver and potentially some glycogenolysis and gluconeogenesis from the kidneys.
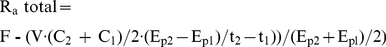
(2)

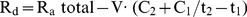
(3)Where F is the infusion rate; E_pl_ and E_p2_ are the [^2^H_2_] glucose enrichments in plasma at time-points *t*_1_ and *t*_2_, respectively; C_1_ and C_2_ are glucose concentrations at *t*_1_ and *t*_2_, respectively; and V is volume of distribution in 160 mL·kg^−1^.

The [U-^13^C] glucose tracer added to each beverage was used to calculate the R_a_ of glucose from the gut. The R_a_ of [^13^C] glucose (R_a_ gut) into plasma was determined by transposition of the Steele equation and the known ^13^C enrichment of the ingested glucose [46] adapted for use with stable isotopes [47].
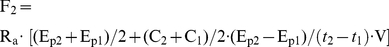
(4)Where F_2_ is the R_a_ of [^13^C] glucose in the blood; R_a_ is the previously determined total R_a_ of glucose (*Eq*. 2). Knowing the R_a_ of [^13^C] glucose in the blood, one can determine the absorption rate of glucose from the gut from the known enrichment of the ingested glucose.
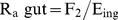
(5)Where R_a_ gut is the R_a_ of gut-derived glucose and E_ing_ is the ^13^C enrichment of the ingested glucose. The rate of endogenous glucose (EGP) was calculated as the difference between R_a_ total and R_a_ gut.
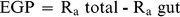
(6)R_a_, R_d_, R_a_ gut and EGP were converted to g.min^−1^ for graphical representation ( = µmol·kg^−1^·min^−1^×kg×180.2/10^−6^).

### Statistical Analysis

A between-subject repeated measures design was utilized for the current study. Exercise variables, blood analytes, plasma enrichment and Western blot data were analyzed using a two-way ANOVA with repeated measures (treatment×time) to determine differences between each condition across time. When a significant main effect or interaction was identified, data were subsequently analyzed using a Bonferroni post hoc test. Plasma glucose and insulin concentrations over the 120 min OGTT were calculated as area under the curve (AUC). Within-group changes over time; glucose kinetics and blood analyte AUC data were checked for statistical significance using one-way repeated-measures ANOVA. All statistical tests were analyzed using statistical package for social sciences (SPSS) version 18.0 (Illinois, Chicago, U.S). Significance for all analyses was set at *P*<0.05. All values are presented as means ± standard error of the mean (SEM).

## Results

### Dietary intake

Dietary analysis indicated that daily energy intake and macronutrient composition of the diet was similar for CON, EX and EX+PRO (Table 2). Thus, the contents of the standardized diet provided prior to and during Day 1 of the study were similar for all groups.

### Exercise variables

Leg-press and leg-extension 1RM values determined during pre-testing were not different between groups (Table 1). Based on the measured 1RM, the resistance lifted on the leg-press machine during Day 1 was 150±17 and 156±17 kg and for EX and EX+PRO, respectively (*P*>0.05). Leg-extension resistance was set at 85±11 and 84±9 kg for EX and EX+PRO, respectively (*P*>0.05). All participants were able to complete the leg-press exercise without reducing the weight. Six participants (four from EX and two from EX+PRO) were unable to complete the leg-extension exercise at the desired resistance, which was then lowered by 2.5–5 kg to enable participants to complete ten repetitions. However, the total exercise volume performed for EX and EX+PRO was not different (Table 1).

### Plasma glucose

**Figure 2:**
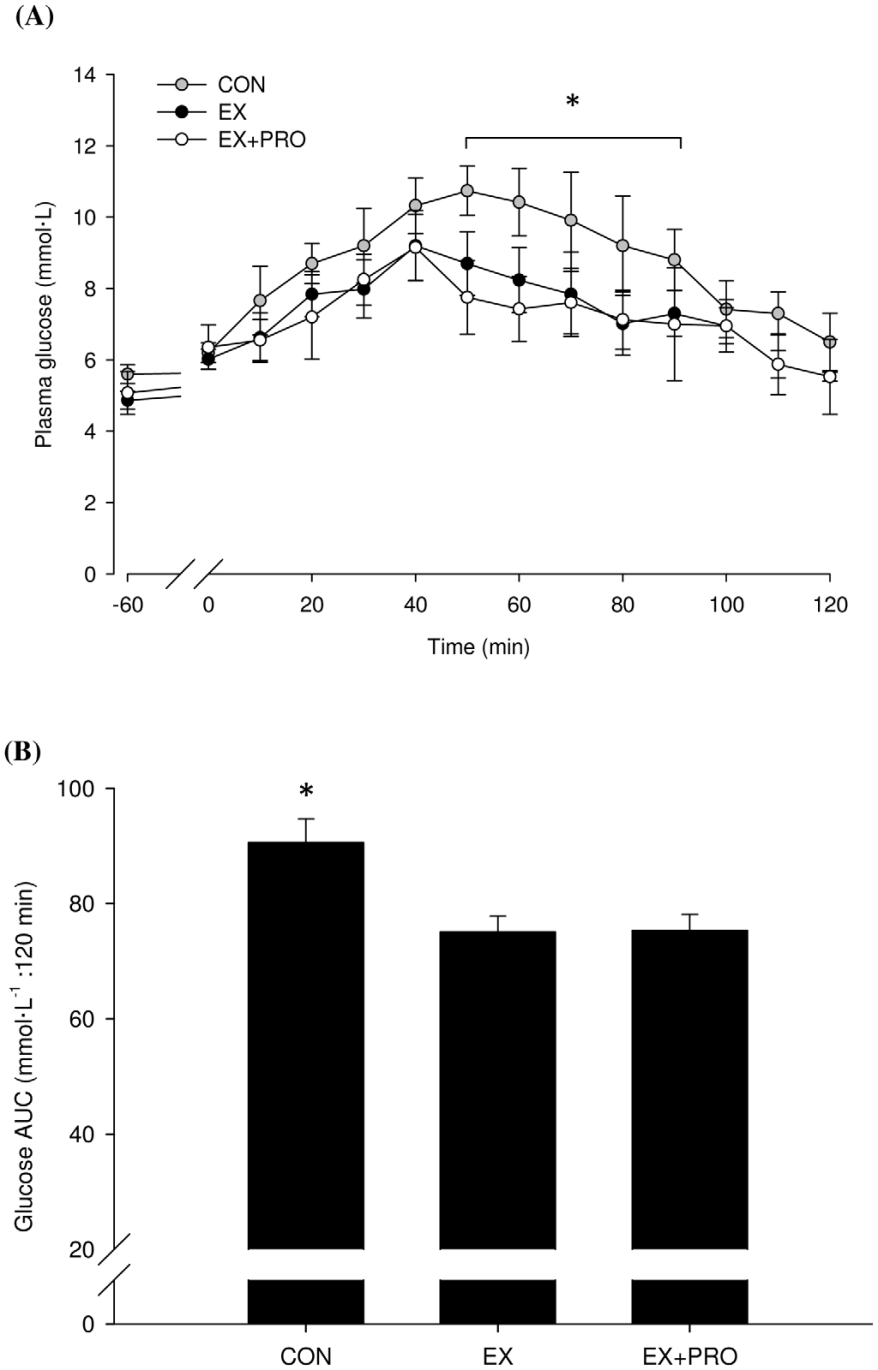
Plasma glucose concentration (A) and AUC (B) during OGTT 24 h following resistance exercise in untrained volunteers. Groups as per Table 1. Values are means ±SEM; *n* = 8 per group. *: significantly greater glucose concentration/AUC for CON compared with EX and EX+PRO (*P*<0.05).

Fasting plasma glucose (5.1±0.4, 5.3±0.7 and 5.7±0.2 mmol·L^−1^ for CON, EX and EX+PRO, respectively) were in the normal range. Basal plasma glucose concentrations were similar on Day 2 (24 h post-exercise) for all groups (5.6±0.3, 4.9±0.3 and 5.1±0.6 mmol·L^−1^ for CON, EX and EX+PRO, respectively). During the OGTT, plasma glucose concentration increased in all groups, peaking 30–50 min after feeding (*P*<0.05; Figure 2A). Plasma glucose peaked at 92±7, 88±9 and 80±8% above basal fasted values for CON, EX and EX+PRO, respectively. Following the peak, plasma glucose concentration decreased, such that by 120 min post-OGTT plasma glucose concentration had returned to basal fasted values. Plasma glucose AUC during OGTT was 17±3% lower for EX and EX+PRO (*P* = 0.02 for both) compared with CON (Figure 2B).

### Plasma insulin

**Figure 3:**
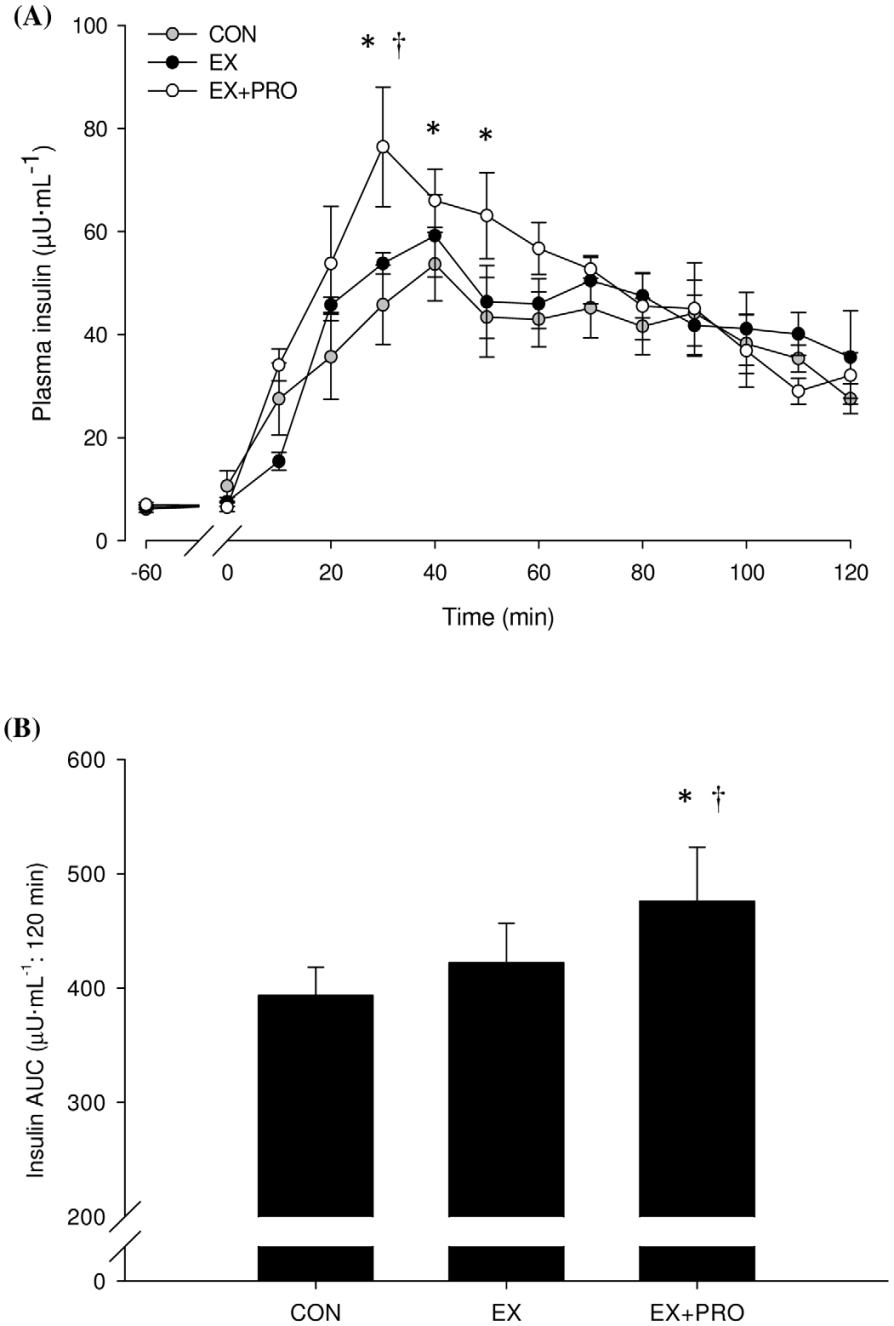
Plasma insulin concentration (A) and AUC (B) during OGTT 24 h following resistance exercise in untrained volunteers. Groups as per Table 1. Values are means ±SEM; *n* = 8 per group. *: significantly greater insulin concentration/AUC than CON (*P*<0.05). †: significantly greater insulin concentration than EX. (*P*<0.05).

Fasting plasma insulin concentrations (7.2±0.6, 5.9±0.7 and 7.1±0.6 µU·ml^−1^ for CON, EX and EX+PRO, respectively) were in the normal range. Basal plasma insulin concentrations were similar on Day 2 for all groups (6.2±0.7, 6.5±0.4 and 6.9±0.5 µU·ml^−1^ for CON, EX and EX+PRO, respectively). Plasma insulin concentrations during OGTT increased by 8.6-, 9.1- and 11.1-fold above basal fasted values for CON, EX and EX+PRO, respectively peaking 30–40 min after feeding (Figure 3A). Plasma insulin AUC during OGTT was significantly greater for EX+PRO compared with EX (*P* = 0.04) and CON (*P* = 0.01). There was no difference in insulin AUC between EX and CON (Figure 3B).

#### Insulin sensitivity

HOMA-(IR) index on Day 1 was 1.55±0.13, 1.69±0.32 and 1.51±0.12 for CON, EX and EX+PRO, respectively. HOMA-(IR) index was not different on Day 2 (1.46±0.17, 1.5±0.12 and 1.64±0.17 for CON, EX and EX+PRO, respectively). Post-prandial insulin sensitivity, calculated using the Matsuda ISI, was greater for EX and EX+PRO (6.95±0.5 and 6.82±0.41, respectively) compared with CON (6.21±0.72; *P*<0.05). There was no difference in Matsuda ISI between EX and EX+PRO.

### Glucose tracer kinetics

**Figure 4:**
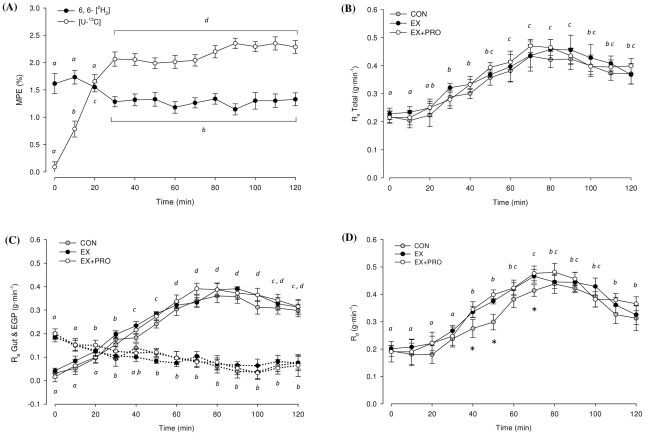
Tracer enrichments and glucose kinetics over 120 min OGTT. **(A) Enrichment of 6, 6-[^2^H_2_] and [U-^13^C] glucose in plasma.** Average for all 3 groups presented, *n* = 24. MPE (%): mole percent excess. (B) Total rate of glucose appearance in plasma (R_a_). (C) Contribution of exogenous glucose appearing from the gut (R_a_ gut) and endogenous glucose production (EGP) to the R_a_ Total; solid lines indicate R_a_ gut, dashed arrows indicate EGP. (D) Rate of glucose disappearance from plasma (R_d_). Means with different subscripts are significantly different from each other. *: significantly lower R_d_ for CON compared with EX and EX+PRO. Values are means ±SEM; *n* = 8 per group.

Plasma enrichment of infused 6, 6-[_2_H^2^] and ingested [U-^13^C] glucose are presented in Figure 4A. R_a_ total, R_a_ gut, EGP and R_d_ over time are presented in Figure 4 (B, C, D, respectively). Average plasma glucose tracer kinetics are presented in Table 3. In all groups, plasma glucose R_a_ total increased over time (*P*<0.05), peaking 70–90 min after feeding (Figure 4B). There was no difference in the plasma glucose R_a_ total between groups. Glucose R_a_ gut demonstrated an increasing contribution to R_a_ total with time (*P*<0.05), whereas EGP demonstrated a reduced contribution to R_a_ total with time (*P*<0.05; Figure 4C). The increase in R_a_ gut peaked 70–90 min after feeding, whereas the decline in EGP reached a nadir 90–100 min after feeding. Glucose R_d_ increased over time in all groups (*P*<0.05; Figure 4D). Glucose R_d_ increased by 127±13, 131±15 and 150±18% above basal values for CON, EX and EX+PRO, respectively. Glucose R_d_ was significantly lower for CON 40–70 min after feeding compared with EX and EX+PRO (*P*<0.05). Average glucose R_d_ and whole-body glucose disposal, (R_d_ expressed as % of R_a_ total) was significantly lower for CON compared with EX and EX+PRO (*P*<0.01; Table 3). The time taken for R_d_ to match the R_a_ total was greater for CON than EX and EX+PRO (*P*<0.05). There was no difference in average glucose tracer kinetics between EX and EX+PRO.

**Table 3:** Plasma glucose kinetics for the 3 groups during OGTT.

	CON (*n* = 8)	EX (n = 8)	EX+PRO (n = 8)
**R_a_ total (g·min^−1^)**	0.34±0.02	0.36±0.02	0.36±0.03
**R_a_ gut (g·min^−1^)**	0.24±0.03	0.26±0.03	0.26±0.04
**Exogenous contribution (R_a_ gut as % of R_a_)**	65.7±6	68.5±6	65.9±7
**EGP (g·min^−1^)**	0.1±0.01	0.1±0.01	0.1±0.01
**Endogenous contribution (EGP as % of R_a_)**	34.2±7	31.4±7	33.9±7
**R_d_ (g·min^−1^)**	0.31±0.02*	0.35±0.02	0.35±0.02
**Glucose disposal (R_d_ as % of R_a_)**	89.4±1.7*	94.1±0.5	95.9±0.6
**Time for R_d_ to match R_a_ (min)**	59±8*	42±5	45±6
**Ingested glucose appearance (%)**	38.4±7.1	41.6±7.6	42.2±7.8

Data presented for [6, 6- ^2^H_2_] glucose rate of appearance (R_a_ total) and disappearance (R_d_) and R_d_ expressed as % of R_a_. Contribution of exogenous [U-^13^C] glucose from the gut (R_a_ Gut) and endogenous glucose production (EGP) to R_a_ total are presented. Groups as per Table 1.

*indicates significantly lower than EX and EX+PRO (*P*<0.05). Values are presented as means ± SEM over 120 min OGTT.

### Muscle glycogen

**Figure 5:**
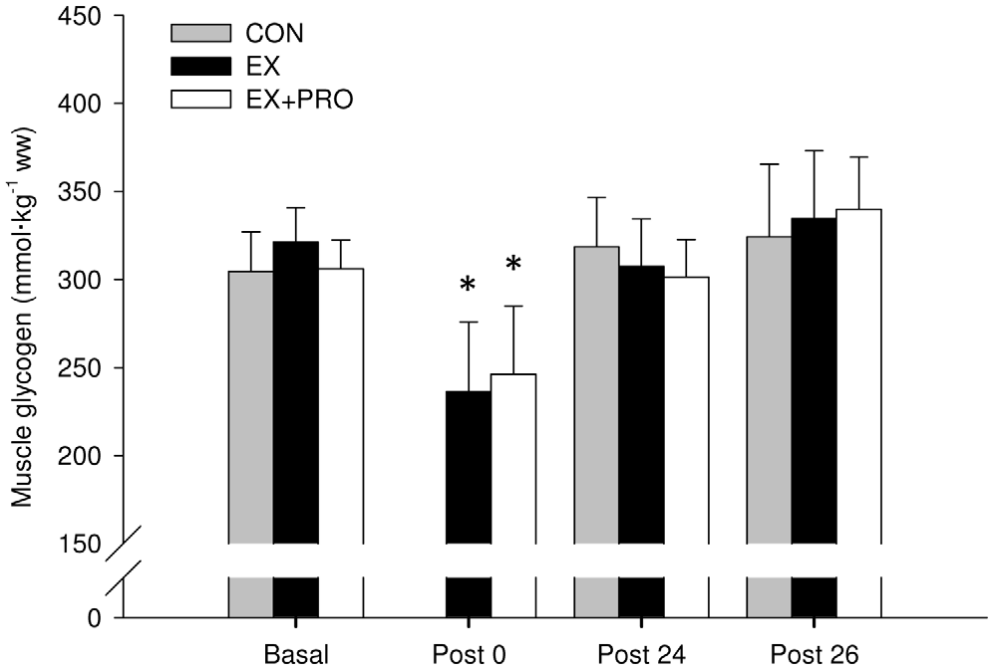
Muscle glycogen content. Values obtained at basal, immediately post-exercise (Post 0), 24 h post-exercise (Post 24) and immediately following OGTT (Post 26). Groups as per Table 1. Values are means ± SEM; *n* = 6 per group. *: significantly lower compared with basal (*P*<0.05).

Basal muscle glycogen concentration was similar for all groups (Figure 5). Immediately post-exercise, muscle glycogen concentration was 26±8 and 19±6% lower for EX and EX+PRO (*P*<0.05), with no difference between groups. Muscle glycogen concentration at 24 h post-exercise had returned to basal values for all groups. There was no significant change in muscle glycogen concentration following OGTT (∼26 h post-exercise), compared with 24 h post-exercise. The absolute change in muscle glycogen content (Table 4) was greater for EX and EX+PRO compared to CON when measured immediately post-RE-to-24 h post-RE (*P*<0.01) and during OGTT from 24 h-to-26 h post-RE (*P*<0.05).

**Table 4:** Absolute change in muscle glycogen.

	CON (*n* = 6)	EX (n = 6)	EX+PRO (n = 6)
**Basal – Post 0**	–	−84.9±−21.9	−59.6±−17.1
**Post 0–Post 24**	13.9±5.7	71.1±22.4**	54.9±15.2**
**Post 24–Post 26**	5.8±3.2	27.1±11.6*	38.6±18.4*

Groups as per Table 1. Change from basal to immediately post-exercise (Basal – Post 0), immediately post-exercise to 24 h post-exercise (Post 0–Post 24) and 24 h post-exercise to 26 h post-exercise (Post 24–Post 26). No change in glycogen assumed for CON from Basal – Post 0. Values are presented as means ± SEM (mmol.kg^−1^.ww).

*Significantly different from CON during the same time period (*P*<0.05).

**Significantly different from CON during the same time period and (*P*<0.01).

### Protein phosphorylation

**Figure 6:**
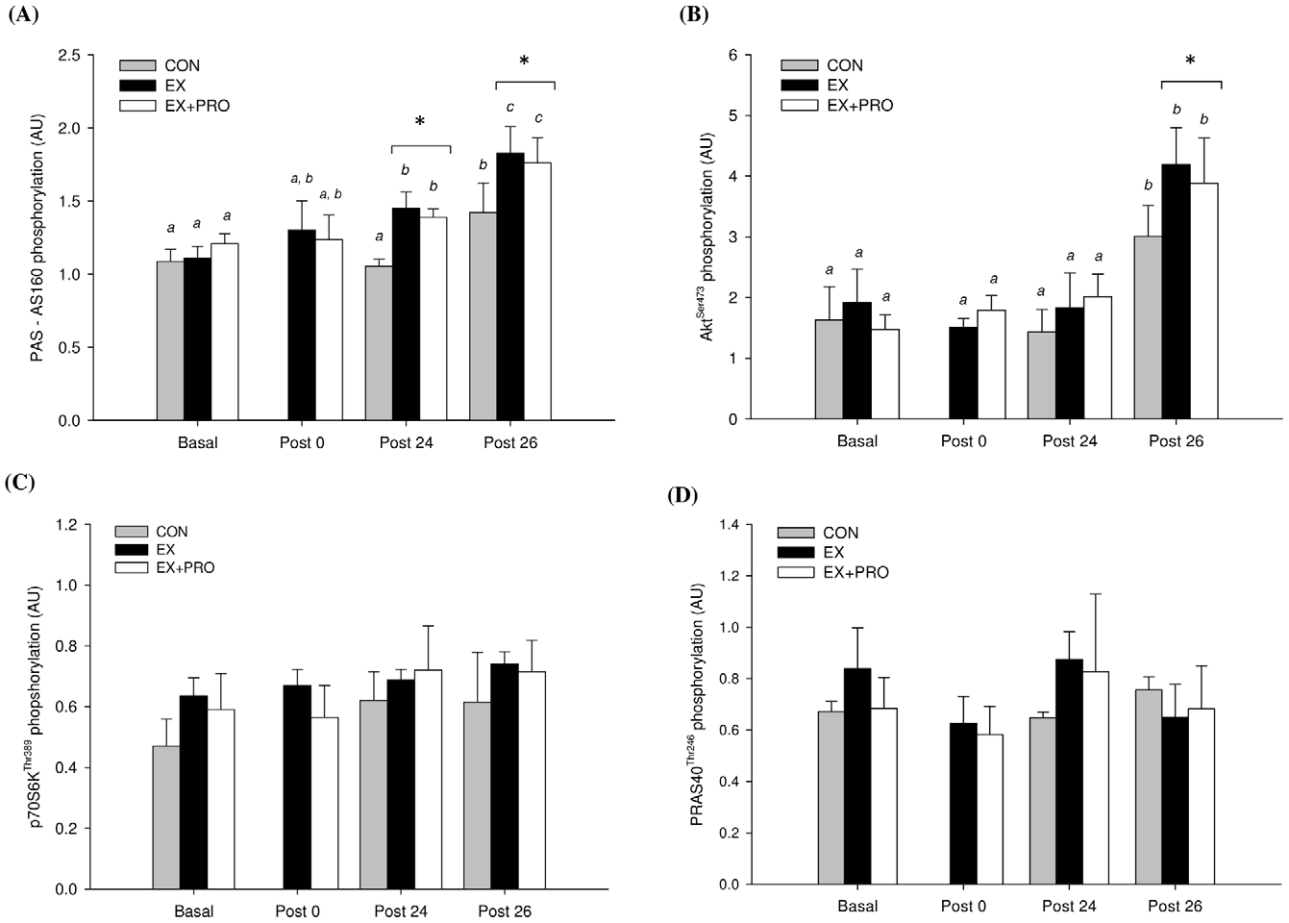
Signalling protein phosphorylation of (A) Akt^ser473^, (B) PAS-AS160/TBC1D4, (C) p70S6K^Thr389^ and (D) PRAS40^Thr246^ from muscle samples taken at 4 different time points. Groups as per Table 1. Values are means ± SEM; *n* = 6 per group. Means with different letters are significantly different from each other (*P*<0.05). *: significantly lower phosphorylation for CON compared with EX and EX+PRO (*P*<0.05).

**Figure 7:**
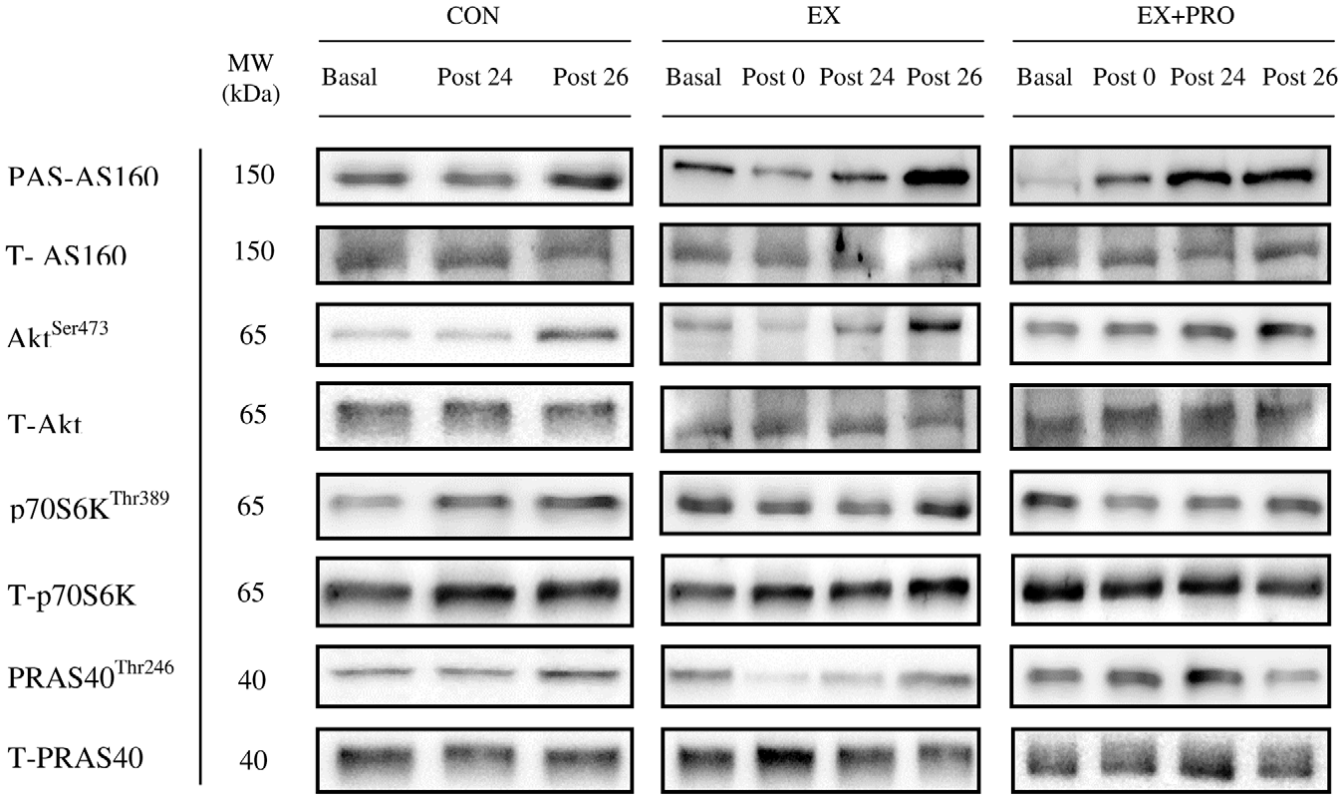
Representative protein phosphorylation blots. Proteins phosphorylation was studied in the basal, fasted state (Basal), immediately post-exercise (Post 0), 24 h post-exercise (Post 24) and 26 h post-exercise following the OGTT (Post 26) and normalized to total protein content.

Basal PAS-AS160/TBC1D4 phosphorylation (Day 1) was similar for all groups (Figure 6A). Compared with basal, AS160/TBC1D4 phosphorylation did not change immediately post-exercise but was increased at 24 h post-exercise for EX and EX+PRO only (*P*<0.05) and was greater compared with CON (*P*<0.01). At 26 h post-exercise, following OGTT, AS160/TBC1D4 phosphorylation increased by 1.4-fold for CON and 1.3-fold for EX and EX+PRO compared with 24 h post-exercise (*P*<0.05) and was greater compared with CON (*P*<0.05). Basal Akt^ser473^ phosphorylation (Day 1) was similar for all groups (Figure 6B). Compared with basal, Akt phosphorylation did not change immediately post- and 24 h post-exercise in all groups. At 26 h post-exercise, following OGTT, Akt phosphorylation increased by 2.2-, 2.3 and 1.9-fold for CON, EX and EX+PRO, respectively, compared with 24 h post-exercise (*P*<0.05). Akt phosphorylation at 26 h post-exercise was greater for EX and EX+PRO compared with CON (*P*<0.05). Basal p70S6K^Thr389^ and PRAS40^Thr246^ phosphorylation (Day 1) was similar for all groups (Figure 6C and 6D, respectively). Compared with basal, p70S6K and PRAS40 phosphorylation was not different immediately-, 24 h- or 26 h-post exercise. There was no between-group difference in p70S6K or PRAS40 phosphorylation immediately-, 24 h- or 26 h-post exercise. All representative western blot images are presented in Figure 7.

## Discussion

The present study expands on previous investigations [17], [20], [48] by providing a physiological mechanism as to how a single bout of intense, lower-body resistance exercise (RE) improves insulin sensitivity in healthy, normoglycemic adults. We demonstrate that prior RE suppresses plasma glucose excursions during an oral glucose tolerance test (OGTT) by increasing the rate of glucose disposal from the circulation. The improvement in insulin-stimulated glucose disposal with prior RE can be attributed in part, to a greater insulin-stimulated phosphorylation of Akt and greater basal and insulin-stimulated phosphorylation of AS160/TBC1D4. However, contrary to our initial hypothesis, co-ingesting protein during OGTT did not augment the response of plasma glucose, insulin sensitivity or glucose disposal to resistance exercise despite a greater insulin response.

To date, studies investigating the acute effect of RE on insulin sensitivity and glucose homeostasis have provided conflicting results [17], [18], [20], [21], [22], [48]. The discrepancies between prior studies may be due, in large part, to methodological differences with the exercise volume or intensity potentially impacting the response of insulin sensitivity to exercise. Prior studies utilizing a relatively low-volume whole-body RE protocol [18], [21] showed no effect on plasma glucose in healthy young [18], [22] and insulin resistant populations [18], [21]. On the other hand, Koopman et al. [17] demonstrate that a lower-limb RE protocol with total volume ∼3-fold greater than the aforementioned studies [18], [21], is sufficient to improve insulin sensitivity in healthy adults. Recent results also suggest that both the intensity and volume of RE impact glucose control in individuals with impaired fasting glucose [19]. Taken together, these findings suggest that higher exercise volumes may be necessary to elicit improvements in insulin sensitivity following RE. Moreover, RE clearly represents an alternative to endurance-type exercise [13], [14], [15], [16] as an effective means of improving glycemic control.

The 17% reduction in plasma glucose response we observed with prior RE is consistent with previous studies [20]. Total glucose appearance and the contribution from endogenous and exogenous sources was not different between groups, indicating that gastric emptying, intestinal uptake and hepatic glucose output were not altered by prior RE or protein co-ingestion during OGTT. However, plasma glucose disappearance/disposal from the circulation did increase with prior RE. Further, we calculated that the time taken for glucose disappearance to match glucose appearance was reduced by ∼37%. The changes in glycogen levels seemingly support this notion. Thus, RE may be a potent intervention to reverse the compromised insulin-stimulated glucose disposal that is prevalent in Type II diabetes [49].

A number of studies have found that prior exercise has no effect on proximal insulin signalling steps, such as Akt phosphorylation [50], [51]. Similarly, we report no effect of RE on basal Akt phosphorylation at 24 h post-exercise. In contrast, phosphorylation of the Rab-GTPase-activating protein AS160/TBC1D4, was greater at 24 h post-exercise. Studies in humans [23], [29] have demonstrated that basal and insulin-stimulated AS160/TBC1D4 phosphorylation is increased several hours post-exercise. Until now, the sustained effect of prior exercise on AS160/TBC1D4 phosphorylation has been demonstrated only in rodent models [31]. Here we show for the first time, in humans, that AS160/TBC1D4 phosphorylation is greater at 24 h post-RE compared with basal and non-exercise values. Moreover, although the insulin-stimulated rise in AS160/TBC1D4 phosphorylation was similar for all groups, the absolute level of AS160/TBC1D4 phosphorylation was greater for EX and EX+PRO compared with CON following the OGTT. Thus, these data support the notion that increased exercise-induced glucose disposal is associated with AS160/TBC1D4 activation. It should be noted however that the small sample size of our experimental groups might have meant that subtle differences in the activation of proteins in the insulin signalling pathway were undetectable.

To our knowledge, this study is the first to examine whether protein co-ingestion augments the response of RE on glycemic control 24 h post-exercise. Under resting conditions, it has been demonstrated that the marked increase in plasma insulin concentrations that prevails when protein and/or amino acids are ingested with carbohydrate can effectively increase glucose disposal [35] and reduce plasma glucose excursions [31], [52]. In our hands, the elevated insulin response with protein co-ingestion did not augment the RE-induced rise in glucose disposal or lower plasma glucose excursions in healthy, normoglycemic individuals. We posit that the lack of a glycemic-lowering effect of additional protein could be attributed to the insulin response to our feeding protocol. Our data reveal that the transient rise in plasma insulin with additional protein was greater between 30–50 min post-feeding (21 and 13% greater than CON and EX, respectively) but was no longer evident by 60 min. In contrast, studies that report a glucose lowering effect with protein co-ingestion have favoured frequent feeding of small boluses to promote a sustained rise in plasma insulin, over several hours; much greater than the present study [31], [35], [52], [53]. Thus, this relatively brief period of hyperinsulinemia may have been insufficient to further lower plasma glucose excursions. However, we chose a single bolus feed to provide a closer representation of the physiological effects of a typical meal, albeit, we acknowledge, one containing more glucose than would usually be consumed by Type II diabetics. The impact of more frequent protein feeding has not been assessed following resistance exercise.

Finally, it is worth mentioning that the studies discussed above, reporting a glucose-lowering effect of protein co-ingestion were conducted under resting conditions [31], [35], [52], [53]. In contrast, we report no additive effect of protein co-ingestion, perhaps due to the fact that the glucoregulatory effects of feeding were assessed following high-intensity RE in healthy, insulin-sensitive participants. Thus, it is possible that the RE stimulus, in healthy adults, is sufficient to promote skeletal muscle glucose uptake to ‘optimal’ levels, beyond which the addition of protein confers no further benefit. Further, our skeletal muscle signalling data indicate that the increased AS160/TBC1D4 phosphorylation following RE was not augmented by additional protein and a greater insulin response. Based on our data, it is unclear whether protein ingestion would augment the glucose-lowering effect of prior RE in Type II diabetics, in whom insulin secretion and glucose transport are impaired.

In conclusion, we have shown that high-intensity resistance exercise improves insulin sensitivity and increases the rate of postprandial glucose disposal, which subsequently lowers post-prandial glucose excursions in healthy, normoglycemic adults. Whereas these data are positive, we acknowledge that such exercise volumes may not be feasible for all insulin resistant/type II diabetic patients. To date, the minimum RE dose required to counteract symptoms of metabolic disease has not been determined and certainly warrants further investigation.
